# Machine Learning Based Analysis of Human Serum *N-*glycome Alterations to Follow up Lung Tumor Surgery

**DOI:** 10.3390/cancers12123700

**Published:** 2020-12-09

**Authors:** Brigitta Mészáros, Gábor Járvás, Renáta Kun, Miklós Szabó, Eszter Csánky, János Abonyi, András Guttman

**Affiliations:** 1 Horváth Csaba Memorial Laboratory of Bioseparation Sciences, Research Center for Molecular Medicine, Doctoral School of Molecular Medicine, Faculty of Medicine, University of Debrecen, 4032 Debrecen, Hungary; brigitta.meszaros@mukki.richem.hu (B.M.); renata.kun@mukki.richem.hu (R.K.); guttman@mik.uni-pannon.hu (A.G.); 2 Research Institute of Biomolecular and Chemical Engineering, University of Pannonia, 8200 Veszprem, Hungary; 3 Department of Pulmonology, Semmelweis Hospital, 3526 Miskolc, Hungary; drszabomiklos@bazmkorhaz.hu (M.S.); ecsanky@bazmkorhaz.hu (E.C.); 4 Complex Systems Monitoring Research Group, University of Pannonia, 8200 Veszprem, Hungary; abonyij@fmt.uni-pannon.hu

**Keywords:** lung cancer, *N-*glycans, machine learning, capillary electrophoresis, surgery

## Abstract

**Simple Summary:**

Globally, there were around 2.1 million lung cancer cases and 1.8 million deaths in 2018. Hungary—where this study was carried out—had the highest rate of lung cancer in the same year. We developed a new analytical method which can be readily used to follow up the tumor surgery by investigating the glycan (sugar) structures of proteins. As the results of such investigations are very complex, computer-assisted machine learning methods were utilized for data interpretation.

**Abstract:**

The human serum *N-*glycome is a valuable source of biomarkers for malignant diseases, already utilized in multiple studies. In this paper, the *N-*glycosylation changes in human serum proteins were analyzed after surgical lung tumor resection. Seventeen lung cancer patients were involved in this study and the *N-*glycosylation pattern of their serum samples was analyzed before and after the surgery using capillary electrophoresis separation with laser-induced fluorescent detection. The relative peak areas of 21 *N-*glycans were evaluated from the acquired electropherograms using machine learning-based data analysis. Individual glycans as well as their subclasses were taken into account during the course of evaluation. For the data analysis, both discrete (e.g., smoker or not) and continuous (e.g., age of the patient) clinical parameters were compared against the alterations in these 21 *N*-linked carbohydrate structures. The classification tree analysis resulted in a panel of *N-*glycans, which could be used to follow up on the effects of lung tumor surgical resection.

## 1. Introduction

Lung cancer is the leading cause of cancer mortality in men and the second in women worldwide, with around a 19% five-year survival rate [1,2]. This low rate is caused, among other factors, by late-stage diagnosis and ineffective treatment follow-up [3]. Early diagnosis improves the survival statistics as treatments can be more focused and effective. Current treatment protocols suggest surgical resection, chemotherapy, biological therapy and radiotherapy, administered alone or in combination [4]. It is essential to understand the pathomechanism of lung cancer in order to explore new diagnostic and therapeutic markers. Lung cancer tumor markers include special molecules produced by the tumor cells and found in various body fluids such as blood, urine, etc. Genetic mutation detection can also be used as a general purpose preventive lung cancer diagnostic method [5]. 

The *N-*glycoprotein profile of the human serum could be altered due to cancer and is usually type-specific [6]. Asparagine-linked protein glycosylation has fundamental roles in molecular and cell biology mechanisms, such as cell communication processes, tumor cell dissociation and invasion, interactions between cell and matrix, tumor angiogenesis, immune modulation and metastasis formation [7]. Therefore, modification of the *N-*glycan profile is one of the hallmarks of malignancy and possibly also holds information about the tumor behavior [8]; thus, it is a promising method to pursue for biomarker discovery. In our earlier work, we suggested a panel of asparagine-linked carbohydrates for lung cancer diagnosis even at early stages by identifying slight changes in the human serum *N-*glycan profile [9].

Several analytical methods are currently available for *N-*glycome analysis, including ultra- or high-pressure liquid chromatography with fluorescence or mass spectrometric detection [7,10], capillary electrophoresis [11], lectin microarrays [12,13] and NMR [14]. The most commonly used high-performance liquid phase separation methods for *N-*glycosylation analysis are capillary electrophoresis and HILIC, often hyphenated with mass spectrometric detection. Capillary electrophoresis separation has several advantages compared to LC, such as short separation times, high efficiency, ultra-low sample volume requirement (in the range of femtoliter and nanoliter injected) and minimal buffer usage, just to list the most important ones [15]. *N-*glycans could be analyzed from solid tissues [16], plasma [17], serum [18], saliva [19] and from FFPE [20], among others. Tissue biopsy samples are more specific than serum, but their collection is rather invasive and the subsequent analytical sample preparation is very complex. Despite the difficulties in tissue sample collection and handling, *N-*glycome profile analysis has also been reported [21,22]. Lattová et al. investigated the *N-*glycome profiles of tissue samples from patients with lung cancer. Their results revealed substantial differences in *N-*glycan distribution associated with the disease compared between cancerous and healthy samples [21]. Lebrilla’s group studied the difference between cancerous and healthy lung tissue samples in order to gain deeper insight into the underlying biological mechanisms of aberrant glycosylation in lung cancer [22]. However, it is difficult to collect tissue samples because most lung cancer patients are frequently diagnosed late, and they are not suitable for surgery or bronchoscopy. Besides lung biopsy, serum samples can be also analyzed to explore any possible changes in the *N-*glycan profile. Such sample analysis is dubbed liquid biopsy and considered to be a non-invasive method. Liquid biopsy can be used for the analysis of *N-*glycome profile alterations caused by malignant transformation [23]. Türe and coworkers compared human serum samples from lung cancer patients and healthy volunteers and concluded that the total serum sialic acid level was significantly elevated due to lung cancer [24]. Moreover, Ruhaak et al. determined the predictive value of serum glycans to distinguish non-small-cell lung cancer cases from controls and identified twelve glycans as significant discriminators [25]. 

Although cancer biomarker discovery is in the spotlight of cutting-edge medical research, our understanding of cancer pathophysiology and pathogenesis is still hampered due to its complexity. Systematic studies of multivariable clinical factors vs. molecular omics data may shed light on important correlations, but deeper relationships cannot be detected without sophisticated computational support. Computational approaches utilize, among others, machine learning, network-based methods, clustering, feature extraction and transformation and factorization [26]. Most often, machine learning (ML) techniques aim at classifying patients into cancer subtypes [27,28,29], supporting therapy decision-making. On the other hand, ML was utilized for biomarker discovery as well. Leclercq et al. reported the identification of biomarker signatures in omics molecular profiling using ML models [30]. More specifically, ML was used for high-throughput glycan profiling by the Rudd group for the interpretation of large-scale quantitative data [31]. Furthermore, ML can assist the glycan biomarker validation in the clinical environment [5,32]. Others developed a machine learning tool (Aristotle Classifier) for discriminating disease states using a panel of glycan features [33].

In this paper, we analyzed the *N-*glycosylation of human serum samples of 17 lung cancer patients before and after surgical resection. Capillary electrophoresis with laser-induced fluorescent detection was used for the serum *N-*glycome profiling. Based on the evaluation of the acquired electropherograms, relative peak areas of 21 *N-*glycans were compared before and after tumor removal and machine learning-based data analysis was performed to explore any possible relationship between clinical parameters and the change in the abundance of certain asparagine-linked carbohydrates.

## 2. Materials and Methods

### 2.1. Chemicals and Reagents

Sodium dodecyl sulfate (SDS) and Nonidet P-40 were from VWR (Radnor, PA, USA). Water (HPLC grade), acetonitrile, tetrahydrofuran (THF), sodium cyanoborohydride (1 M in THF), glycerol, dithiothreitol (DTT) and acetic acid were obtained from Sigma Aldrich (St. Louis, MO, USA). The Fast Glycan Labeling and Analysis Kit was from SCIEX (Brea, CA, USA). The endoglycosidase PNGase F was from Asparia Glycomics, (San Sebastian, Spain). 

### 2.2. Sample Preparation

All serum samples were collected with the appropriate ethical permissions (approval number: 23580-1/2015/EKU (0180/15)) and informed patient consent at the Department of Pulmonology in the Semmelweis Hospital (Miskolc, Hungary). Serum samples were taken from 17 lung cancer patients before and after tumor removal surgeries; see details in Table 1. Sample preparation protocol included denaturation, glycan release, fluorophore labeling and magnetic bead-mediated cleanup. Serum samples were diluted a hundredfold by HPLC-grade water, followed by denaturation at 70 °C for 10 min by adding 2.0 µL denaturation solution of the Fast Glycan kit (SCIEX). Glycan release was performed by the addition of 1.0 µL of PNGase F enzyme (200 mU) to the reaction mixture and incubated at 60 °C for 20 min to ensure complete deglycosylation. The endoglycosidase digestion reaction was stopped by the addition of the labeling solution containing 1.0 µL of 40 mM 8-aminopyrene-1,3,6-trisulfonic acid (APTS) in HPLC-grade water, 2.0 µL of NaBH_3_CN (1 M in THF), 10 µL 50% acetic acid, 8.0 µL THF. The reaction mixture was incubated in a heating block overnight at 37 °C. After the labeling step, the samples were purified by magnetic beads following the Fast Glycan Sample Preparation and Analysis protocol and analyzed by capillary electrophoresis utilizing laser-induced fluorescent detection. 

### 2.3. Capillary Electrophoresis

Glycan analysis was performed by capillary electrophoresis utilizing laser-induced fluorescent detection (CE-LIF) using a PA800 Plus Pharmaceutical Analysis System (SCIEX). All CE measurements were accomplished in 40 cm effective length (50 cm total length), 50 µm ID bare-fused silica capillaries filled with the HR-NCHO separation gel buffer (SCIEX) in triplicate. Then, 30 kV electric potential was applied during the separation step in reversed polarity mode (cathode at the injection side, anode at the detection side) at 30 °C. A two-stage sample injection was used: 1) 1.0 psi for 5.0 s water pre-injection, 2) 2.0 kV for 2.0 s sample injection. Data collection and analysis were carried out by the 32 Karat (version 10.1) software package (SCIEX). Relative percentage area values of the separated peaks were calculated by the PeakFit v4.12 software SeaSolve Software Inc. (San Jose, CA, USA).

### 2.4. Data Analysis

Changes in the relative peak area values were assessed to investigate the relationship between the relative peak area alterations of the glycan structures and the clinical outcome. For the data analysis, both discrete (e.g., smoker or not) and continuous (e.g., age of the patient) clinical data were utilized as independent variables. Clinical data, which were earlier reported as potential risk factors of lung cancer, were selected from the patients’ anamnesis for the current study, following the guidance of [34]. Logically, discrete variables were treated as integers 0 or 1, i.e., if the patient was a smoker, the variable has a value of 1; otherwise, 0. All selected discrete clinical data were transformed into matrix form. The discrete variables were estimated by classification tree analysis using integrated MathWorks Matlab (Natick, MA, USA) functions. In order to avoid model overtraining, the datasets were divided into two sections: one for teaching and one for controlling the behavior. The accuracy of the model was checked by cross-validation and evaluated based on the fraction of the correctly classified data. Thus, accuracy = 1 would represent the case when all the samples were correctly classified. Please note, in the current study, the acceptance threshold was set as 0.63 to ensure reliable prediction. Cases above this threshold were evaluated, while below, they were neglected. The involved discrete variables were: smoker (smoking or stopped smoking earlier), comorbidities or lack of comorbidities with diabetes, atherosclerosis, chronic obstructive pulmonary disease (COPD), tumor type (squamous cell carcinoma, adenocarcinoma, anaplasticus-cell carcinoma, small-cell neuroendocrine carcinoma), the outcome of the surgery, i.e., positive or negative (successful tumor removal and recovered patient or unsuccessful resection when tumor relapsed within two years), as well as other frequently occurring diseases (lipoma, hyperlipidaemia, spondylosis, arthritis, struma nodosa or osteoporosis comorbidity or not). As the available number of eligible patients was limited and the investigated factors were numerous, additional independent variables, e.g., clinical parameters, were generated by pairing the individual parameters. For example, COPD comorbidity with atherosclerosis was considered as an additional independent variable. The complexity of the decision tree was not restricted to a certain depth but was inherently determined by the variables. 

Linear regression models were used to find correlations between the so-called continuous clinical variables and the change in relative peak areas of the *N-*glycans monitored. Continuous variables included the age of the patient, years of smoking, blood glucose level, tumor status, C-reactive protein (CRP) level, number of smoked cigarettes over lifetime. The optimal model was evaluated by stepwise regression with the threshold specified as 0.005.

## 3. Results and Discussion

**Figure 1:**
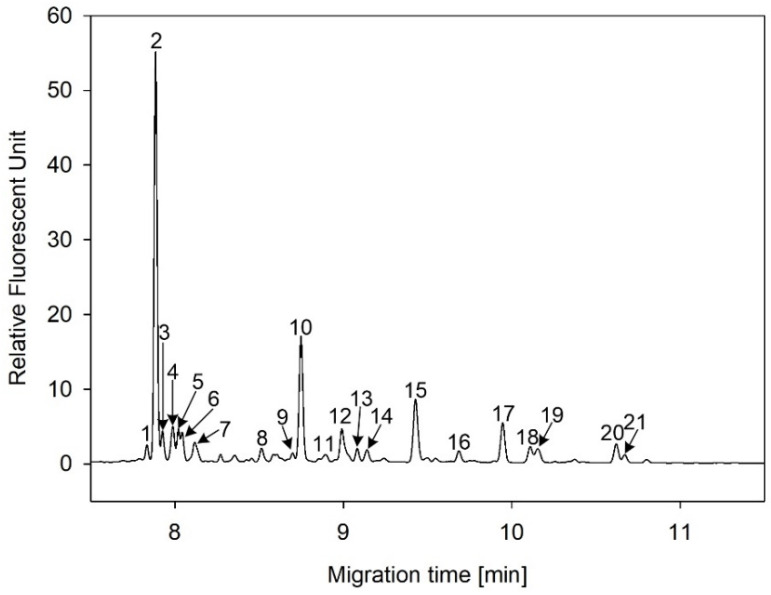
Capillary electrophoresis analysis of PNGase F released and APTS-labeled *N-*glycans from pooled healthy human serum. Peaks with >1% relative abundance are numbered. Separation conditions: 40 cm effective capillary length (50 cm total length), 50 μm ID bare-fused silica; 30 kV (0.17 min ramp time) separation voltage in reversed polarity mode. LIF detection (excitation: 488 nm/emission: 520 nm); separation temperature 30 °C. Injection: water preinjection 5.0 s at 1.0 psi, followed by 2.0 kV/2.0 s sample.

In this study, serum samples from 17 lung cancer patients were investigated before and after tumor-removing surgery. First, as reported earlier [9], a pooled control serum *N-*glycome was profiled by capillary electrophoresis with laser-induced fluorescence detection and the resulting electropherogram is shown in Figure 1. Glycans with greater than 1% relative peak area were selected for downstream multiparametric analysis. The selected 21 peaks fulfilling this criterion are numbered in Table 2 with their names and structures, following the Oxford notation [35].

Alterations in the relative peak areas of the 21 selected glycan structures were evaluated by discrete and continuous machine learning (ML) analysis to find correlations between clinical parameters and the changes in the individual quantity of the glycans of interest. Classification tree analysis is one of the predictive modeling approaches used in ML. In total, 51 clinical variables (including original/individual ones as well as generated/paired ones) were considered in the classification tree analysis. Table 3 summarizes the correlations between certain clinical parameters and the corresponding alterations in the *N-*glycan structure, where the accuracy of the model was above the previously set threshold of 0.63. Our results revealed that smokers and smokers with successful surgery had changes in peak #15 (co-migrating FA2 and M6). If the patients were smokers at the time of sampling, the relative peak area of peak #15 increased by 11.35% with 0.75 accuracy. 

Besides smoking, if the outcome of the surgery was positive, the relative peak area increment of peak #15 (FA2 + M6) was equal or greater than 16.14% with 0.88 accuracy. Lung cancer comorbidity with other diseases except diabetes, atherosclerosis or COPD, and non-smokers having no atherosclerosis, resulted in the increment of the relative peak area of the peak #21 (M9) greater or equal than 35% with 0.75 accuracy and in the increment greater or equal than 17.8% with 0.81 accuracy, respectively. If the operation had a positive outcome, it showed the decrement of the relative peak area of peak #2 (A2G2S(6)2) equal or less than 38.1%, and of peak #6 (FA2G2S2) where the change was less than 13.58% with 0.69 accuracy. The change in the amount of peak #6 (FA2G2S2) was equal to or greater than 31.67% with 0.69 accuracy in patients having COPD besides positive outcome of surgery. Smoker patients with positive surgery outcome showed a slight alteration in the amount of peak #2 (A2G2S(6)2) and peak #3 (FA3G3S(6)3) structures: the relative peak area of peak #2 (A2G2S(6)2) increased equally to or higher than 9.34% or its increment was lower than 9.34% together with at least 3.96% relative peak area increment of peak #3 (FA3G3S(6)3) with 0.63 accuracy. Moreover, COPD without atherosclerosis caused equivalent to or higher than 10.97% increment of the relative peak area of peak #12 (FA2G2S1) with 0.81 accuracy. However, COPD comorbidity with atherosclerosis induced the change in the relative peak area of peak #1 (FA4BG4[3,3,3,3]S4) with at least 18.51% increment; otherwise, the increment was less than 18.51% together with higher than 28.15% decrement of the relative peak area of peak #20 (FA2G2) with 0.63 accuracy. For patients without atherosclerosis whose surgery was successful, the relative peak area of peak #5 (A2BG2S2) decreased by more than 19.10%, or the relative peak area of peak #2 (A2G2S(6)2) increment was at least 9.34% with 0.63 accuracy. Equal to or greater than 35.24% increment with 0.88 accuracy of relative peak area of peak #7 (FA2BG2S2 co-migrating with FA3G3S(3)3) was measured for patients having no atherosclerosis and failed tumor removal surgery. Lung cancer comorbidity with atherosclerosis generated the change in the relative peak area of the following glycan structures: peak #5 (A2BG2S2) decreased 19.10% and the relative peak area of peak #2 (A2G2S(6)2) changed by a maximum 6.06% with 0.63 accuracy. In addition, patients with atherosclerosis and positive surgery showed that the increment of peak #11 (A2BG2S1) relative peak area was higher than 18.37% with 0.75 accuracy. Nonetheless, in case of smoker patients with unsuccessful operation, the relative peak area of peak #11 (A2BG2S1) increased more than 19.26% with 0.81 accuracy. Although the patient stopped smoking earlier and the operation failed, the *N*-glycome profile changed as follows: the relative peak area of peak #4 (A2G2S(3)2) decreased by more than 28.26% or peak #4 (A2G2S(3)2) decrement was higher than 28.26% together with the decrease in the relative peak area of peak #18 (FA2[3]G1) by at least 18.79% with 0.69 accuracy. Lung cancer patients with comorbidity with COPD whose surgery was unsuccessful showed alteration of the relative peak area of peak #3 (FA3G3S(6)3) and peak #13 (FA2BG2S1). The relative area of peak #3 (FA3G3S(6)3) decreased by more than 34.01% or peak #3 decrement (FA3G3S(6)3) was greater than 34.01% together with the increment of the relative peak area of peak #13 (FA2BG2S1) at least 19.26% with 0.69 accuracy.

In this study, the selected 21 glycan structures were grouped into the following subclasses: total afucosylated (afucosylated and high mannose), total sialylated, total terminal galactosylated and neutral glycan (all glycans with no sialylation) structure subclasses. Similarly to individual glycan classification tree analysis, 51 clinical variables were involved in the glycan subclasses investigation. The results of the machine learning analysis are summarized in Table 4 with their corresponding accuracy values. Only results with at least 0.63 accuracy were taken into consideration. Less than 21% relative peak area decrease was observed after failed surgery of non-smokers with 0.69 accuracy; or those without COPD with 0.69 accuracy, or those with atherosclerosis with 0.75 accuracy; or diabetes with 0.75 accuracy; those without atherosclerosis but with diabetes with 0.81 accuracy, smokers with diabetes with 0.81 accuracy; or those without atherosclerosis with 0.69 accuracy or those without COPD with accuracy 0.69; those without COPD but with diabetes with 0.75 accuracy. However, positive outcome of the surgery with 0.69 accuracy or non-smoker without diabetes with 0.63 accuracy clinical parameters induced the decrement of the relative peak area of total afucosylated subclass of greater than 21%. The clinical parameter of diabetes without COPD resulted a range of change of 1.4–4.57% in the relative peak area of the total afucosylated subclass with 0.63 accuracy. The relative peak area of the neutral glycan subclass was changed for the smoking group with positive surgery outcome; its decrement was greater than 9.26% with accuracy 0.63. Two clinical parameters induced alteration of the relative peak area of the total sialylated glycan class: non-smoker with positive outcome of surgery and atherosclerosis without diabetes. Atherosclerosis without diabetes increased the relative peak area of the total sialylated glycan class by at least 7.41% with 0.75 accuracy; however, non-smoker with positive outcome of surgery decreased it by more than 1.05% with 0.81 accuracy. Lung cancer comorbidity with diabetes influenced the relative peak area of the total terminal galactosylated glycan subclass; its increment was at least 81.53% with 0.81 accuracy. Lung cancer comorbidity with other diseases (such as lipoma, hyperlipidaemia, spondylosis, arthritis, struma nodosa, osteoporosis) also increased the relative peak area of the total terminal galactosylated glycan class by at least 81.53%, or if the change was less than 81.53%, then the relative peak area of total sialylated glycan class increased by at least 9.07% with 0.69 accuracy. Besides lung cancer, COPD comorbidity with atherosclerosis impacted the relative peak area of the total sialylated and total afucosylated subclasses. This disease combination increased the amount of relative peak area of the total sialylated glycan subclass by at least 7.4%, or if the decrement of the relative peak area of the total sialylated subclass was less than 7.4%, the decrement of the total fucosylated glycan subclass was between 5.56% and 2.21% with 0.63 accuracy. Non-smoker patients with atherosclerosis showed similar behavior as COPD with atherosclerosis.

The results of the evaluation of the linear regression analysis (see Table 5) showed that the number of years of smoking, the age of the patient, the blood glucose unit, CRP value, the lung cancer stage and the number of smoked cigarettes have linear correlations with the alterations in the relative peak area of *N*-glycans, with satisfying R^2^ values. Equations (1)–(6) represent the linear regression models, which describe the relationship of the clinical parameters with the change in the relative peak area of *N*-glycans. Equation (1) shows that the years of smoking contributes to the change in the relative peak areas of the following *N*-glycans: FA2G2S1, FA2[6]G1 and M7, FA2[3]G1, FA2B[6]G1 and M8, FA2G2 and M9 (peaks #12 #17–21). Moreover, the status of lung cancer alters the relative peak areas of A4G4S(6)2, FA2[6]G1 and M7, FA2B[6]G1 and M8, FA2G2 and M9 (peaks #14 #17 #19–21) according to Equation (3). The age of the patient, the number of smoked cigarettes, blood sugar value and CRP value cause changes in the relative peak area of FA2[6]G1 and M7, FA2[3]G1, FA2B[6]G1 and M8, FA2G2 and M9 (peaks #17–21) based on Equations (2) and (4)–(6), respectively.

## 4. Conclusions

Based on the machine learning analysis described in this paper, novel information was gained regarding the relationship between certain clinical variables and the change in the relative peak areas of serum *N-*glycan structures. Positive outcome of the surgery showed a significant correlation with *N-*glycan change for even smoker or non-smoker patients and either atherosclerosis or without atherosclerosis. Moreover, negative outcome of the operation also for smoker or non-smoker patients can be related to the change in the relative peak area of certain *N-*glycan structures (FA2BG2S1, A2G2S(3)2, FA2[3]G1). Negative outcome of the surgery with COPD or without atherosclerosis has a relationship with the change in the relative peak area of FA3G3S(6)3 and FA2BG2S1 or FA2BG2S2 and FA3G3S(3)3. This study also demonstrated that lung cancer comorbid with COPD also has a significant correlation, even with or without atherosclerosis, with the alteration in the relative peak area of FA4BG4S(3)4 and FA2G2 or FA2G2S1. An even more significant increment was observed in the relative peak area of FA2G2S2 due to positive outcome of surgery in the case of lung cancer patients with COPD. In the *N-*glycan profile of the human serum of lung cancer non-smoker patients without atherosclerosis, the surgical resection increased the relative peak area of the M9 glycan structure.

Besides evaluation of the relationship between the clinical parameters and the relative peak area changes in the individual *N-*glycan structures, correlations of the relative peak area alterations of *N-*glycan groups with the clinical parameters were also found. The most significant clinical parameters causing alterations in the relative peak area of total afucosylated subclasses before and after surgery were identified (Table 4). Clinical parameters such as non-smoker who has atherosclerosis and COPD comorbid with atherosclerosis have correlations with both total afucosylated and total sialylated in the same way. However, non-smoker whose surgery has positive outcome or does not have diabetes but atherosclerosis showed a change in the relative peak area of total sialylated. Moreover, if the patient has another disease comorbid with lung cancer, besides the change in relative peak area of total sialylated, the amount of total terminal galactosylated glycans also altered with significant accuracy. Lung cancer comorbid with diabetes generated a change in the relative peak area of the total terminal galactosylated subclass. Only one parameter (positive outcome of the surgery and smoker) from the 51 caused a change with satisfying accuracy in the relative peak area of neutral glycans. The effect of continuous clinical parameters on the *N-*glycan profile was evaluated using linear regression analysis. The results suggest a linear correlation between serum *N-*glycome alterations caused by lung tumor surgery and continuous clinical parameters. Thus, by utilizing the identified correlations, a panel of *N-*glycans can be assembled to follow up on the surgical resection. Based on the results reported in this paper, we plan to carry out future studies involving a larger cohort of participants for statistical purposes. Furthermore, we plan to apply the demonstrated workflow to monitor the effects of chemotherapy on the *N-*glycan profile.

## Figures and Tables

**Table 1:** Clinical patient information.

Patient #	Age	Sex	Ethnicity	Histology	Stadium
1	78	male	Caucasian	squamous cell carcinoma	I/b
2	70	male	Caucasian	small-cell neuroendocrine carcinoma	I/a
3	61	female	Caucasian	adenocarcinoma	II/b
4	79	female	Caucasian	adenocarcinoma	I/b
5	52	male	Caucasian	adenocarcinoma	III/b
6	63	male	Caucasian	squamous cell carcinoma	II/b
7	68	female	Caucasian	adenocarcinoma	I/a
8	53	male	Caucasian	adenocarcinoma	I/a
9	58	female	Caucasian	adenocarcinoma	I/a
10	75	male	Caucasian	adenocarcinoma	III/a
11	66	female	Caucasian	adenocarcinoma	I/a
12	61	male	Caucasian	anaplasticus-cell carcinoma	II/b
13	63	female	Caucasian	adenocarcinoma	I/a
14	70	male	Caucasian	adenocarcinoma	I/a
15	68	male	Caucasian	squamous cell carcinoma	II/a
16	75	male	Caucasian	adenocarcinoma	I/a
17	77	male	Caucasian	adenocarcinoma	I/a
Age average: 66.8, Age median: 68, Age range: 52–79					

**Table 2:** *N-*glycan structures in the control pooled human serum sample. Peaks with >1% relative abundance are listed.

Peak Notation	Structures	Glycan Structures
1	FA4BG4S(3)4	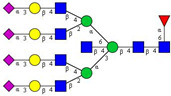
2	A2G2S(6)2	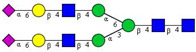
3	FA3G3S(6)3	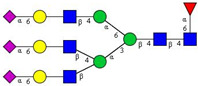
4	A2G2S(3)2	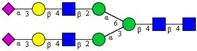
5	A2BG2S2	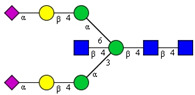
6	FA2G2S2	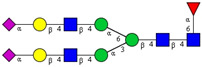
7	FA2BG2S2, FA3G3S(3)3	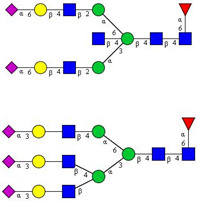
8	FA2[6]G1S1	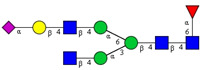
9	A3G3S(3)2	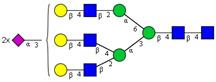
10	A2G2S(6)1	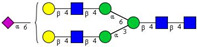
11	A2BG2S1	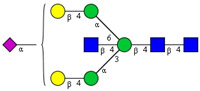
12	FA2G2S1	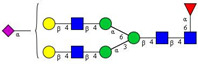
13	FA2BG2S1,M5	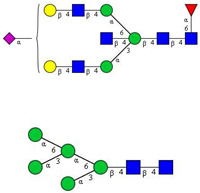
14	A4G4S(6)2	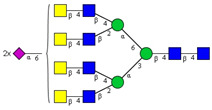
15	FA2, M6	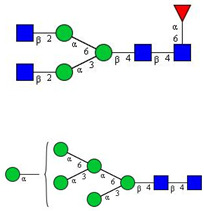
16	FA2B	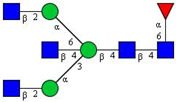
17	FA2[6]G1, M7	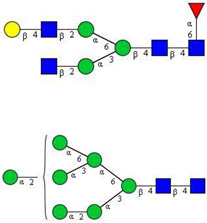
18	FA2[3]G1	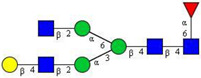
19	FA2B [6]G1, M8	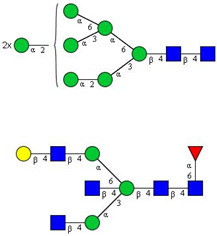
20	FA2G2	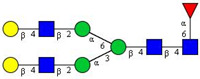
21	M9	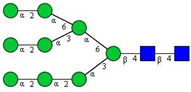

**Table 3:** Results of the classification tree analysis showing the relationship between clinical characteristics and the change in the relative peak area of the *N-*glycan structures with their respective accuracy.

Clinical Parameters	Results	Accuracy
Positive outcome of the surgery	ΔA2G2S(6)2 ≥ −38.1 and ΔFA2G2S2 < 13.58	0.69
Smoker	ΔFA2, M6 < 11.35	0.75
Have atherosclerosis	ΔA2BG2S2 ≥ −19.10 and ΔA2G2S(6)2 < 6.06	0.63
Have other disease	ΔM9 ≥ 35	0.75
Positive outcome of the surgery and smoker	ΔA2G2S(6)2 ≥ 9.34 or ΔA2G2S(6)2 < 9.34 and ΔFA3G3S(6)3 ≥ 3.96	0.63
Positive outcome of the surgery and non-smoker	ΔFA2, M6 ≥ 16.14	0.88
Negative outcome of the surgery and smoker	ΔFA2BG2S1 ≥ 19.26	0.81
Negative outcome of the surgery and non-smoker	ΔA2G2S(3)2 < −28.26 or ΔA2G2S(3)2 ≥ −28.26 and ΔFA2[3]G1 < −18.79	0.69
Negative outcome of the surgery and have COPD	ΔFA3G3S(6)3 < −34.01 or ΔFA3G3S(6)3 ≥ −34.01 and ΔFA2BG2S1 ≥ 19.26	0.69
Positive outcome of the surgery and have atherosclerosis	ΔA2BG2S1 ≥ 18.37	0.75
Positive outcome of the surgery and not have atherosclerosis	ΔA2BG2S2 < −19.10 or ΔA2BG2S2 ≥ −19.10 and ΔA2G2S(6)2 ≥ 9.34	0.63
Negative outcome of the surgery and not have atherosclerosis	ΔFA2BG2S2, FA3G3S(3)3 ≥ 35.24	0.88
Have COPD and atherosclerosis	ΔFA4BG4[3,3,3,3]S4 ≥ 18.51 or ΔFA4BG4[3,3,3,3]S4 < 18.51 and ΔFA2G2 < −28.15	0.63
Have COPD and not have atherosclerosis	ΔFA2G2S1 ≥ 10.97	0.81
Positive outcome of the surgery and have COPD	ΔFA2G2S2 ≥ 31.67	0.69
Non-smoker and not have atherosclerosis	ΔM9 ≥ 17.80	0.81

**Table 4:** Results of the classification tree analysis showing the relationship between clinical characteristics and the change in the relative peak area of the *N-*glycan subclasses with their respective accuracy values.

Clinical Parameters	Results	Accuracy
Positive outcome of the surgery	Δ total afucosylated ≥ −21%	0.69
Have diabetes	Δ total terminal galactosylated ≥ 81.53%	0.81
Have other disease	Δ total terminal galactosylated ≥ 81.53% or Δ total terminal galactosylated < 81.53% and Δ total sialylated ≥ 9.065%	0.69
Positive outcome of the surgery and smoker	Δ neutral < −9.26%	0.63
Positive outcome of the surgery and non-smoker	Δ total sialylated < −1.05%	0.81
Negative outcome of the surgery and non-smoker	Δ total afucosylated < −21%	0.7
Negative outcome of the surgery and no COPD	Δ total afucosylated < −21%	0.69
Negative outcome of the surgery and atherosclerosis	Δ total afucosylated < −21%	0.75
Have COPD and atherosclerosis	Δ total sialylated ≥ 7.4% or Δ total sialylated < 7.4% and −5.56% ≤ Δ total afucosylated < −2.21%	0.63
Not have COPD but diabetes	Δ total afucosylated < −21%	0.75
Not have COPD and diabetes	Δ total afucosylated ≥ 4.57% or Δ total afucosylated < 1.4%	0.63
Negative outcome of the surgery and have diabetes	Δ total afucosylated < −21%	0.75
Not have diabetes but atherosclerosis	Δ total sialylated ≥ 7.41%	0.75
Not have atherosclerosis but diabetes	Δ total afucosylated < −21%	0.81
Smoker and have diabetes	Δ total afucosylated < −21%	0.81
Non-smoker and not have diabetes	Δ total afucosylated ≥ −21	0.63
Smoker and not have COPD	Δ total afucosylated < −21	0.69
Non-smoker and have atherosclerosis	Δ total sialylated ≥ 7.4% or Δ total sialylated < 7.4% and −5.56% ≤ Δ total afucosylated <−2.21%	0.63
Smoker and not have atherosclerosis	Δ total afucosylated < −21%	0.69

**Table 5:** The suggested regression models for the analysis of the relationship between continuous variables (i.e., continuous clinical parameters) and the change in the relative peak area of the *N-*glycan structures with their respective squared regression coefficient. Please note, the annotation of independent variables follows the nomenclature of Table 2, i.e., *x*12 stands for the alteration of relative peak area of peak #12.

Formalization of the Relationship	R^2^	#
y~−6.31+1.81·x12±0.16·x17·x18+0.76·x17·x19+(−0.90)·x17·x20+0.03·x17·x21+(−0.38)·x18·x19+0.74·x18·x20+(−0.03)·x20·x21	0.99	Equation (1)
y~48.64+0.34·x21+0.22·x17·x19+(−0.25)·x17·x20+(−0.14)·x18·x19+0.17·x18·x20+0.02·x19·x20	0.95	Equation (2)
y~2.32+0.05·x14+0.001·x17·x20+0.001·x19·x21	0.81	Equation (3)
y~397250+1688·x17·x18+(−1408)·x17·x19+(−1623)·x18·x20+393·x18·x21+832·x19·x20	0.77	Equation (4)
y~5.79+0.01·x17·x18+(−0.01)·x17·x19+0.005·x17·x21+(−0.01)·x18·x19+0.01·x18·x20+(−0.01)·x18·x21+0.01·x19·x20+0.005·x20·x21	0.99	Equation (5)
y~18.8+(−0.04)·x17·x19+0.04·x17·x21+(−0.05)·x18·x21+0.04·x19·x20	0.96	Equation (6)
